# Facile Discovery of a Diverse Panel of Anti-Ebola Virus Antibodies by Immune Repertoire Mining

**DOI:** 10.1038/srep13926

**Published:** 2015-09-10

**Authors:** Bo Wang, Christien A. Kluwe, Oana I. Lungu, Brandon J. DeKosky, Scott A. Kerr, Erik L. Johnson, Jiwon Jung, Alec B. Rezigh, Sean M. Carroll, Ann N. Reyes, Janelle R. Bentz, Itamar Villanueva, Amy L. Altman, Robert A. Davey, Andrew D. Ellington, George Georgiou

**Affiliations:** 1 Department of Chemical Engineering, University of Texas at Austin, Austin, Texas, USA; 2 Department of Molecular Biosciences, University of Texas at Austin, Austin, Texas, USA; 3 Department of Virology and Immunology, Texas Biomedical Research Institute, San Antonio, TX, USA; 4 Department of Biodefense and Protein Diagnostics, Luminex Corporation, Austin, TX, USA; 5 Center for Systems and Synthetic Biology University of Texas at Austin, Austin, TX, USA; 6 Institute for Cell and Molecular Biology, University of Texas at Austin, Austin, TX, USA; 7 Department of Biomedical Engineering, University of Texas at Austin, Austin, Texas, USA

## Abstract

The ongoing evolution of Ebolaviruses poses significant challenges to the development of immunodiagnostics for detecting emergent viral variants. There is a critical need for the discovery of monoclonal antibodies with distinct affinities and specificities for different Ebolaviruses. We developed an efficient technology for the rapid discovery of a plethora of antigen-specific monoclonal antibodies from immunized animals by mining the V_H_:V_L_ paired antibody repertoire encoded by highly expanded B cells in the draining popliteal lymph node (PLN). This approach requires neither screening nor selection for antigen-binding. Specifically we show that mouse immunization with Ebola VLPs gives rise to a highly polarized antibody repertoire in CD138^+^ antibody-secreting cells within the PLN. All highly expanded antibody clones (7/7 distinct clones/animal) were expressed recombinantly, and shown to recognize the VLPs used for immunization. Using this approach we obtained diverse panels of antibodies including: (i) antibodies with high affinity towards GP; (ii) antibodies which bound Ebola VLP Kissidougou-C15, the strain circulating in the recent West African outbreak; (iii) non-GP binding antibodies that recognize wild type Sudan or Bundibugyo viruses that have 39% and 37% sequence divergence from Ebola virus, respectively and (iv) antibodies to the Reston virus GP for which no antibodies have been reported.

Ebolaviruses are negative-sense RNA filamentous viruses that cause very high morbidity and mortality^1^. Host cell entry is mediated first by the attachment of the heavily glycosylated glycoprotein (GP) on the viral envelope to the host cell encoded T-cell immunoglobulin and mucin domain 1 (TIM-1)^2^. Following cathepsin cleavage in the lysosome, GP mediates cellular entry by binding the host cell encoded Niemann-Pick C1 (NPC1)^3^. Five antigenically distinct ebolaviruses exhibiting 35–45% genome sequence divergence have been discovered^4^: Ebola virus (abbreviated as EBOV, formerly designated as Zaire ebolavirus); Sudan virus (SUDV); Bundibugyo virus (BDBV); Reston virus (RESTV, for which no zoonotic infections have been reported to date)^5^; and Taï Forest virus (TAFV, one incident of human infection)^6^. The recent EBOV outbreak in West Africa, centered in Guinea, Sierra Leone, and Liberia with isolated outbreaks in Nigeria and Mali, was the largest ever with a mortality rate estimated at 70% of recorded definitive clinical outcomes (http://www.cdc.gov/vhf/ebola/outbreaks/2014-west-africa/index.html)^7^. Phylogenetic comparison of isolates from the recent outbreak^8^ with 20 Ebolavirus genomes from earlier outbreaks suggested that the 2014 West African virus likely spread from central Africa within the past decade, having diverged from a common ancestor around 2004^9^. The five Ebolavirus species have varying rates of molecular evolution, with the highest of 8.21 × 10^−4^ nucleotide substitutions/site/year for Reston virus^10^. The ongoing evolution of Ebolaviruses poses significant challenges to the development of immunodiagnostics. Specifically, there is a critical need for the discovery of panels of monoclonal antibodies with distinct affinities and specificities for different Ebolaviruses.

Antibodies to EBOV and SUDV have been produced from hybridomas^11^,^12^; by *in vitro* screening of synthetic Fab libraries^13^,^14^, and from human immune antibody libraries constructed from infected individuals^15^. However additional monoclonal antibodies to Ebolaviruses are urgently needed both for diagnostic purposes and as therapeutics^16^,^17^. Specifically, the generation of diagnostic antibodies to Ebolaviruses is complicated by the structural complexity of the GP, which is heavily glycosylated in a host cell-specific manner^18^,^19^ and subjected to proteolytic cleavage during entry^20^, as well as by the sequence diversity of the Ebolaviruses. Finally, characterization of useful antibodies to Ebolaviruses is limited by the safety concerns associated with handling the live virus.

Antibody discovery has relied either on the immortalization^21^ or cloning of antibodies isolated from individual B cells obtained from an antigen-challenged host^22^,^23^,^24^,^25^,^26^,^27^ or, alternatively, on the *in vitro* isolation from combinatorial libraries using a variety of screening techniques^28^. The current collection of antibody technologies is predicated on the isolation of clones that display high antigen binding. However, animal immunization induces the stimulation and expansion of a highly diverse population of B cells encoding an antibody repertoire with a wide range of antibody affinities^22^,^23^. Antibodies with low affinity nonetheless may exhibit other highly desirable properties, including broad cross-reactivity or heteroclitic specificity, i.e. stronger binding reaction to a different antigen other than the one used for immunization^24^,^25^,^29^. Unfortunately, there is no straightforward way to identify such interesting antibodies. For example, while the isolation of antibodies that bind to multiple antigens (e.g. to different flu hemagglutinins) or that neutralize rapidly evolving pathogens such as HIV-1 or flu has been accomplished by B cell cloning, the process typically requires the screening of many thousands of B cells and therefore is very laborious and expensive^26^,^27^,^30^,^31^.

In order to satisfy the need for a wider variety of antibodies to Ebolaviruses, we developed a novel approach to comprehensively mine the full suite of antibody diversity, shaped by *in vivo* selective mechanisms and generated within the boundary of reactive secondary lymphoid tissues in immunized animals. We reasoned that antibodies encoded by antigen-stimulated B cells that had undergone the greatest degree of expansion within the confinement of a secondary lymphoid organ are most likely to display desirable antigen recognition properties including heteroclite recognition of diverse Ebolaviruses. Briefly, mice were first immunized in the footpad with Ebola virus-like particles (VLPs). Footpad immunization triggers a strong and highly-focused immune reaction in the PLN, especially for particles <40 nm such as VLPs^32^. Antigen experienced, CD138^+^ B cells (plasmablasts) from the PLN were isolated and the natively paired V_H_:V_L_ repertoire encoded by these cells was determined by NextGen sequencing^33^. Antibodies corresponding to the highest frequency V_H_:V_L_ pairs, and thus likely arising from the most clonally expanded and highly-transcribing CD138^+^ B cells within the PLN, were expressed recombinantly and their binding properties were characterized in detail. In each of two mice tested, 7/7 antibodies encoded by the highest frequency antigen-draining PLN plasmablast sequences recognized the antigen (Ebola VLPs) with several binding to recombinant GPs with up to nM affinities. Interestingly, even though animals had been immunized with EBOV VLPs, mining of the expanded native B cell repertoire within the PLN resulted in antibodies that also recognized SUDV and BDBV. In a separate experiment we also employed this technique to generate the first RESTV GP-specific antibodies. Thus, the antibodies reported here collectively constitute a panel of reagents for the detection of most Ebolaviruses.

## Results

### Immunization of the PLN yields antigen-specific antibodies

**Figure 1:**
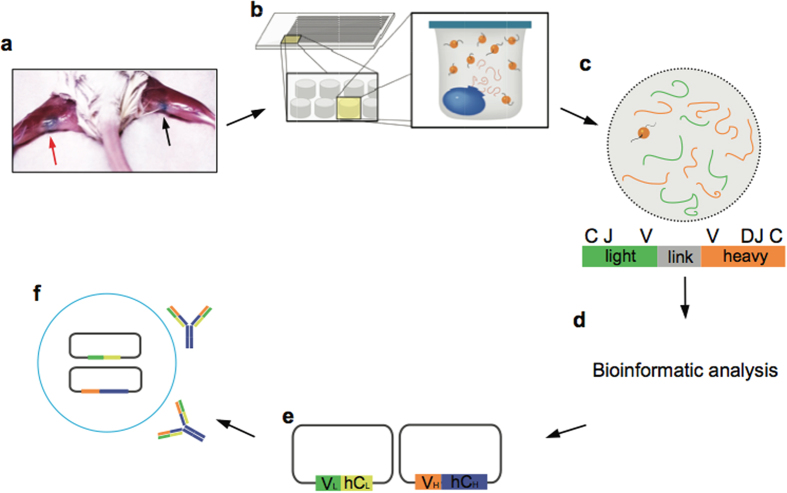
Isolation of antibodies by mining the paired V_H_:V_L_ repertoire of draining popliteal lymph node (PLN) antibody-secreting B cells. (**a**) Footpad immunization leads to a marked increase in cellularity within the ipsilateral popliteal lymph node relative to the contralateral lymph node (red and black arrows respectively). (**b**) PLN CD138^+^ cells isolated by magnetic sorting are deposited into 125 pL wells on PDMS slides that also contain poly(dT) beads. Cells are lysed *in situ* and mRNA is captured on the poly(dT) beads^33^. (**c**) The poly(dT) beads are emulsified and V_H_:V_L_ amplicons are generated following reverse transcription and overlap extension PCR. (**d**) V_H_:V_L_ amplicons are sequenced using Illumina 2 × 250 MiSeq and the highest frequency V_H_:V_L_ pairs are identified via bioinformatics analysis. (**e**) Highest frequency V_H_ (orange) and V_L_ (green) genes are synthesized and cloned into IgH and IgL expression vectors containing human IgG1 (blue) and human kappa (yellow) constant regions, respectively. (**f**) Following co-transfection into Expi293 cells, recombinant IgG antibodies are expressed and purified.

Figure 1 summarizes our approach for mining the antibody repertoire encoded by the most highly expanded, antigen-experienced B cells following antigen stimulation. Footpad immunization leads to a strong inflammatory response in the draining popliteal lymph node (Fig. 1a). Unlike lymph nodes that drain sites of frequent extracorporeal interaction (e.g. the oral cavity or lungs), the germinal centers of the popliteal lymph node are normally relatively unstimulated^34^. Footpad immunization results in a marked increase in cellularity in the ipsilateral popliteal lymph node relative to the unstimulated contralateral lymph node^35^,^36^, and a large fraction of the constituent antibody-secreting B cells were expected to be antigen-specific.

Ebola VLPs were produced by co-transfection of HEK293FT cells with plasmids encoding the three major virus structural proteins: nucleoprotein (NP), VP40 and GP of the EBOV Mayinga strain and were purified by sucrose density centrifugation. Electron microscopy and gel staining confirmed that the VLPs displayed a morphology and consistency characteristic of EBOV virions (Supplementary Fig. 1). Three mice were immunized with Ebola VLPs in emulsified adjuvant in the left hind footpad, followed by boost immunization in the lateral hock to minimize pain and discomfort. An anti-VLP titer of >1:10^4^ was observed after the second boost (Supplementary Fig. 2a) in all three mice, and two mice (denoted as ZM1 and ZM2) were chosen for further analysis. A final boost was administered and 6 days later the popliteal lymph nodes were extracted. As expected, the ispilateral PLN was observed to be hypertrophic (Supplementary Fig. 2b).

CD45R^−^CD19^−^CD138^+^ antibody secreting B cells were enriched by magnetic sorting and the paired V_H_:V_L_ repertoire from single cells was determined following sequestration of the cells into 125 pL wells on PDMS slides^33^ (Fig. 1b). Approximately 1 × 10^5^ CD45R^−^CD19^−^CD138^+^ plasmablasts were isolated by magnetic sorting, of which 2.5–3.5 × 10^4^ cells were processed to create natively paired V_H_:V_L_ amplicons. Linked V_H_:V_L_ amplicons of approximately 850 bp were generated and then sequenced using Illumina MiSeq technology (Fig. 1c). High quality reads were clustered based on the CDRH3:CDRL3 sequences (Fig. 1d).

### The PLN plasmablast IgG repertoire elicited by immunization with EBOV

**Figure 2:**
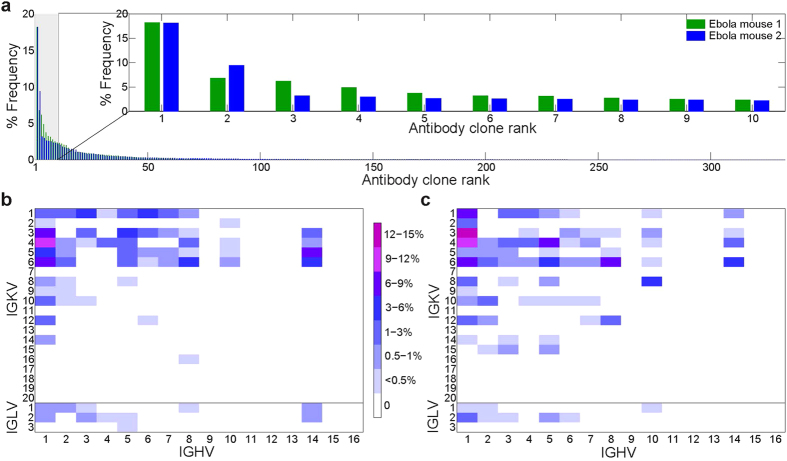
Characteristics of the PLN V_H_:V_L_ repertoire in CD138^+^ antibody secreting cells. (**a**) Polarization of V_H_:V_L_ repertoire after immunization with EBOV VLPs. The frequency of each unique CDRH3:CDRL3 antibody clonotype is shown as a percentage of total sequencing read counts. CDRH3 sequencing reads having at least 96% identity at the nucleotide level were clustered and compiled, then analyzed for corresponding CDRL3 per pair. Sequences identified in <2 reads were excluded to minimize sequencing error^33^. Inset: frequency of the ten most frequently observed CDRH3:CDRL3 antibody clonotypes from each mouse. (**b,c**) V_H_:V_L_ gene family usage of unique CDRH3:CDRL3 clonotypes in mouse ZM1 and ZM2, respectively.

283 and 333 unique V_H_:V_L_ pairs (represented by ≥2 sequence reads per pair each), comprising the PLN plasmablast repertoires were identified in mouse ZM1 and mouse ZM2, respectively. The repertoires from both Ebola VLPs immunized mice investigated were heavily skewed, with the top ten most abundant V_H_:V_L_ pairs representing 53.9% and 48.4% of the total sequence counts in each mouse, respectively (Fig. 2a). We observed a strong bias in germline V-gene usage in immunized mice (Fig. 2b,c). Biased usage of IGHV subgroups 1–3 and 5^37^,^38^, as well as IGKV1, 3, 4 and 6 have been previously observed in the repertoires of unimmunized mice^38^. Likewise, in Ebola VLPs immunized mice, IGHV1, IGHV5, as well as IGKV1, IGKV3, IGKV4, and IGKV6 heavy and light V genes were most strongly represented. Importantly, however, the IGHV8, IGHV14, and IGKV5 families were also strongly overrepresented in both mice immunized with EBOV VLPs. The enrichment of IGHV8 in the plasmablast repertoire from PLN of Ebola VLPs immunized animals is particularly noteworthy as this germline family is expressed at a very low level in mice^37^,^38^ and additionally it was shown to be utilized at very low frequencies in CD138^+^ lymphocytes from animals immunized with various other antigens^22^ (Supplementary Fig. 3a,b).

The repertoire of Ebola VLPs immunized mice displayed an average CDRH3 length distribution similar to that observed in CD138^+^ repertoires previously reported^22^ (Supplementary Fig. 3c,d), although there was a slight skewing toward shorter CDRH3 lengths. Shorter CDRH3s are commonly found among antibodies that bind carbohydrates and thus the skewing observed here likewise may have reflected the elicitation of antibodies to the glycan component of the heavily glycosylated GP^39^. The plasmablast repertoire from EBOV VLPs immunized mice displayed a higher level of somatic hypermutation level in the framework 3 (FR3) heavy chain region compared to plasmablast repertoires reported for mice hyperimmunized with various other protein antigens^22^ (Supplementary Fig. 3e,f).

### Construction and characterization of anti-EBOV VLP antibodies

**Figure 3:**
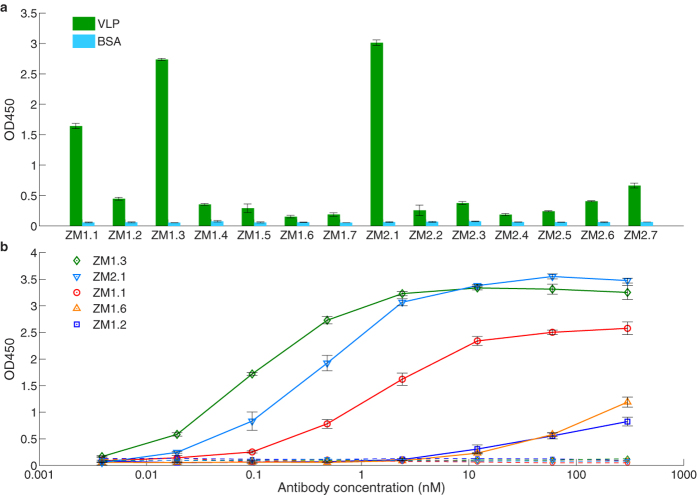
Functional characterization of IgG antibodies isolated via mining of the PLN CD138^+^ B cell repertoire. (**a**) Binding to EBOV VLPs for antibodies encoded by the seven most frequently observed CDRH3:CDRL3 clonotypes from the sequenced PLN CD138^+^ B cell repertoires of each mouse. (**b**) Binding to purified EBOV recombinant GP for select antibodies as determined by ELISA. Binding to BSA as a control is shown in correspondingly colored dashed lines. Error bars represent the standard error of the mean for three technical replicates.

The CDRH3 and CDRL3 amino acid sequences of the 7 highest frequency V_H_:V_L_ clonotypes from each mouse, together with the respective V(D)J gene segments are listed in Table 1. Antibody genes for these 14 most prevalent V_H_:V_L_ antibody clonotypes were synthesized and cloned as mouse V region-human constant domain chimeric antibodies, expressed in HEK293 cells, and characterized for antigen binding (Fig. 1e,f). KZ52, a very well characterized Ebola virus recognizing antibody isolated from a survivor of the 1995 Kikwit outbreak^15^,^40^, was expressed as a positive control. All 14 antibodies gave an ELISA signal above background on plates coated with the immunizing antigen, i.e. EBOV (Mayinga strain) VLPs (Fig. 3a). ELISA titer analysis revealed that the majority of the antibodies bound VLPs at a titer >1:1,000 (Supplementary Table 1). Interestingly, the ELISA signals did not correlate with the frequency of the respective antibodies in the PLN CD138^+^ repertoire.

5/14 antibodies recognized the recombinant uncleaved form of the EBOV Mayinga strain GP (Fig. 3b). One antibody bound to GP with a single-digit nM equilibrium dissociation constant (ZM1.3, K_D_ = 7.7 nM), as determined by SPR analysis (Table 2, Supplementary Fig. 4), while two others (ZM1.1 and ZM2.1) exhibited K_D_ values in the 10 nM range. Finally, 2/5  GP specific IgGs, ZM1.2 and ZM1.6, exhibited lower affinities (K_D_ = 156 and 635 nM, respectively (Table 2, Supplementary Fig. 4)), consistent with the lower titer of those antibodies for Ebola VLPs.

The three highest affinity, EBOV GP-specific antibodies (ZM1.1, ZM1.3 and ZM2.1) were tested for binding to VLPs encoding GPs from Ebola strains isolated from two different outbreaks: 034-KS (Democratic Republic of Congo, 2008, NCBI Accession number HQ613402) and Kissidougou-C15 (Kissidougou, Guinea, 2014, NCBI Accession number KJ660346) (Supplementary Fig. 5). Both ZM1.3 and ZM2.1 showed higher binding to Ebola 034-KS and Kissidougou-C15 VLPs than the well-studied KZ52 antibody (K_D_ for purified Mayinga GP = 1.55 nM) (Supplementary Fig. 6a,b). We observed that KZ52 failed to recognize VLPs containing a Mayinga GP N550K variant in which Arg 550 residue was replaced with Lys, a mutation observed in Marburg GP^41^ (Supplementary Fig. 6c), while ZM1.1, ZM1.3 and ZM2.1 were still able to bind the Mayinga GP N550K variant. This finding indicates that ZM1.1, ZM1.3 and ZM2.1 likely recognize a different epitope than KZ52. Interestingly, ZM1.3 displayed heteroclitic specificity in that it bound better to EBOV 034-KS and Kissidougou-C15 VLPs than to the immunization Mayinga strain VLPs (EC_50_ of 0.334 nM and 0.39 nM, respectively compared to 1.7 nM for Mayinga VLPs). These antibodies also bound to live wild type Ebola virus (Supplementary Fig. 6d), indicating that they should be useful for developing diagnostic assays against primary biological samples.

**Figure 4:**
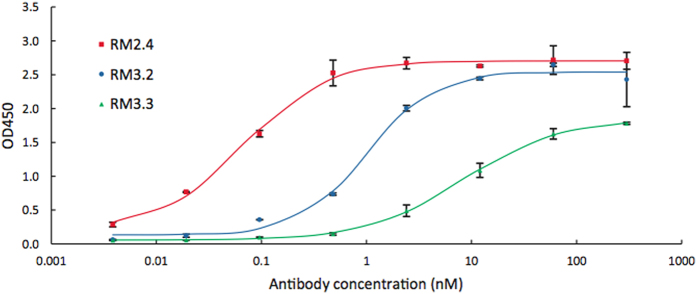
Functional characterization of IgG antibodies isolated via mining of RESTV immunized PLN CD138^+^ B cell repertoire. Binding to RESTV recombinant GP for select antibodies as determined by ELISA. Curves were fitted using 4-parameter logistic non-linear regression. Error bars represent the standard error of the mean for three technical replicates.

Finally, encouraged by the results detailed above, we used the strategy described in Fig. 1 to develop antibodies that recognized Reston Ebola virus (RESTV, Reston, 1996, NCBI Accession number AB050936), an Ebolavirus for which no anti-GP monoclonal antibodies are available. RESTV VLPs were generated, mice were immunized as above, and the repertoire encoded by PLN plasmablasts was determined. We identified three antibodies designated RM2.4, RM3.2, and RM3.3 (Supplementary Table 2) that bound to both RESTV VLPs as well as to RESTV recombinant GP (Fig. 4).

### Diagnostic utility of non-GP binding antibodies

**Figure 5:**
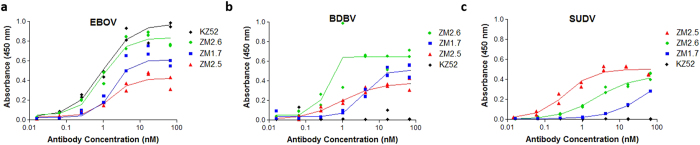
Binding of cross-reactive antibodies isolated via mining of the PLN CD138^+^ B cell repertoire to wild type EBOV, BDBV and SUDV viruses. ELISA assays using antibodies ZM1.7, ZM2.5, ZM2.6, and KZ52 for the detection of (**a**) wild type Ebola virus; (**b**) wild type Bundibugyo virus; and (**c**) wild type Sudan virus. Assays were performed in two technical replicates. Lines represent measurements fitted via 4-parameter logistic nonlinear regression for EC_50_.

While 9/14 anti-EBOV VLP antibodies did not show binding to the GP and thus presumably recognized other VLP proteins (NP, VP40) they nonetheless are of diagnostic utility. Specifically, in addition to binding to EBOV Mayinga VLPs, antibodies ZM1.4, ZM1.7, ZM2.2, ZM2.3, ZM2.5, and ZM2.6 displayed measurable binding to Bundibugyo Virus (BDBV, Bundibugyo, 2007, NCBI Accession number FJ217161), while ZM1.7, ZM2.2, ZM2.5, and ZM2.6 also showed binding activity to Sudan Virus (SUDV, Gulu, 2000, NCBI Accession number AY729654) (Representative ELISA data is shown in Fig. 5a–c, also see Supplementary Table 3). Thus, antibodies elicited by immunization with EBOV VLPs bound differentially to phylogenetically diverse variants.

## Discussion

Here we report a facile and rapid approach for generating large panels of distinct monoclonal antibodies that, unlike existing antibody discovery platforms, does not rely on screening for antigen binding; and further describe application of this approach by developing panels of diagnostic antibodies for Ebola virus strains. We found that in multiple animals immunized with EBOV or RESTV VLPs, the repertoire of CD138^+^ plasmablasts in the draining PLN was dominated by highly expanded, antigen-specific, antibody sequences. We find that these highly expanded sequences include antibodies that display high affinity to GP, bind to live, wild-type virus and, somewhat surprisingly, display diverse specificities to VLPs from different Ebolaviruses. Among the antibodies isolated, six recognized live Bundibugyo or both Bundibugyo and Sudan viruses in addition to EBOV. These results demonstrate the power of mining the antibody repertoire of highly expanded B cells after immunization for discovery of antibodies with interesting properties.

The ability to isolate 7 (and possibly more) distinct antibodies with very different CDR3 sequences per animal as well as a much larger number of somatic variants whose sequences are also available in the V_H_:V_L_ sequence database provides a rich source of antibodies for practical purposes. Undoubtedly, many PLN B cells encoding antigen-specific antibodies are likely to have been subject to more limited expansion and thus are present at a lower abundance within the repertoire. However, such medium or low abundance antibody sequences within the repertoire are present at comparable levels to those elicited by environmental stimuli and thus recognizing unrelated antigens. Therefore the low abundance antigen-specific antibody sequences in the repertoire cannot be identified directly without significant additional effort.

The method we have pioneered should prove particularly useful for the facile development of diagnostic (and possibly therapeutic) antibodies. Starting from antibody-secreting B cells, it took us only 3 weeks to produce and characterize a diverse set of antigen-specific antibodies with a variety of useful specificities. This method should be particularly valuable for assessing and combating fast spreading pandemics.

In case of rapidly spreading emerging diseases such as Ebola, rapid and robust diagnostics are critical for treatment and disease control. For Ebola in particular, validated PCR based assays suitable for field work in third world countries are not available. Highly sensitive antibody based immunodiagnostics are extremely important and easy to implement^42^. However, a dearth of monoclonal antibodies for emergent Ebolaviruses limits the ability to use antigen-capture technologies for viral identification of early-stage infections. The panel of antibodies identified and characterized here should be useful for diagnostic applications including discriminating SUDV, BDBV, and EBOV. By using multiplex immunoassay platforms such as the Luminex MagPlex^®^ technology, it should be possible to multiplex up to 50 different antibodies with varying specificities for a broad range of epitopes, thus ensuring wide coverage of Ebolaviruses variants. Studies to incorporate the antibodies we have described here onto the Luminex MagPlex^®^ diagnostic platform for field applications are on-going.

## Methods

### VLP Production and Characterization

VLPs were produced by co-transfection of HEK293FT (Invitrogen) cells with plasmids encoding the three major virus structural proteins, NP, VP40 and GP. All open reading frames for virus structural proteins were obtained from NCBI and were codon optimized for mammalian cell expression using Gene Designer (DNA 2.0), synthesized (Epoch Life Science) and inserted into either pcDNA3 (for Ebola GP) or pCAGGS (all other genes) mammalian expression plasmids. Sequences of the structural protein ORFs were verified and are available upon request to R. A. D. Cells were transfected with each of 5 μg NP, 5 μg VP40 and 1 μg GP encoding plasmids by the calcium chloride/BES transfection method. After 24 h cells were washed with DMEM, which was replaced with DMEM containing 10% (v/v) FBS. After an additional 24 h, the culture supernatant was collected and clarified by centrifugation at 3750 rpm for 30 min at 4 ^o^C to remove cell debris. The supernatants were then overlaid onto a 5 mL 20% (w/v) sucrose cushion in 20 mM NaCl, 20 mM HEPES, pH 7.4 in an SW28 ultracentrifuge tube (Beckman). The VLPs were then pelleted by centrifugation at 28,000 rpm for 2 h at 4 ^o^C in an SW28 rotor. To further purify VLPs, the pellet was resuspended in PBS and overlaid onto a 20 to 60% sucrose step gradient (5% increments) in 20 mM NaCl, 20 mM HEPES, pH 7.4 in a SW55 rotor tube (Beckman). The gradient was centrifuged at 38,000 rpm for 2 h at 4 ^o^C after which an opaque band corresponding to VLPs was visible. Fractions were collected corresponding to the band as well as directly above and below it and analyzed by SDS-PAGE, staining for total protein with Krypton stain (Thermo Scientific) as well as immunoblotting. The middle fraction containing the peak of the VLPs was stored at −80°C until required. For immunoblotting, proteins were transferred to nitrocellulose membranes and stained using broadly reactive polyclonal antibodies against GP (gift from Dr. Andrew Hayhurst, Texas Biomedical Research Inst.), VP40 (gift from Dr. Ricardo Carrion, Texas Biomedical Research Inst.) or NP (IBT Bioservices). A specific monoclonal antibody against Zaire Ebolavirus GP was also used (4F3, IBT Bioservices). Appropriate secondary antibodies were purchased from LiCor Biosciences. Blots were imaged using a LiCor Odyssey SA imager. Electron microscopy of VLPs was performed at the University of Texas Health Sciences Center, San Antionio, Department of Pathology electron microscopy facility. VLPs were adhered to copper grids. The samples were fixed in glutaraldehyde and osmium tetroxide was used as contrast agent. Images were captured on a Philips 208S digital imaging electron transmission microscope.

### Immunizations and Serum Titer Determination

The study was approved by the University of Texas Institutional Animal Care and Use Committee (AUP-2013–00015). All animal experiments were carried out in accordance with the approved protocol. VLPs in PBS pH 7.4 were emulsified in a 1:1 ratio with TiterMax Gold adjuvant (Sigma). For footpad immunizations, 20 μL (containing a total of 5 μg VLPs) antigen/adjuvant mixture was injected into the subcutaneous space of three BALB/c mice; for lateral hock injections, up to 50 μL was injected into the subcutaneous space just proximal to the lateral aspect of the ankle. Mice were immunized at footpad on day 0 for primary immunizations, and in the lateral hock on days 21, 35, and 77 for secondary immunizations. At days 10, 28, and 42 mice were bled for titration of the antigen-specific response. In order to determine serum antibody titer, mice were restrained in a tube restrainer, the tail wiped with isopropyl alcohol, and small incisions made with a fresh scalpel blade to nick the tail vein. 20–50 μL blood was obtained and allowed to coagulate at room temperature (RT) for 30 min, followed by centrifugation at 13,000 g for 15 min to pellet the clot. The serum was then used for ELISA assays. High binding ELISA plates (Corning) were coated overnight (O/N) at 4°C with 50 μL of 4 μg/mL Zaire Ebolavirus VLPs in PBS pH 7.4. Antigen solution was decanted and plates were then blocked at RT for 2 h in 2% milk (w/v) in PBS. Blocking solution was then decanted and plates were then incubated with 50 μL of serum diluted three-fold from 1:100 to 1:218,700 in 2% milk (w/v) in PBS for 1 h. Plates were then aspirated and washed 3 times with PBS containing 0.05% tween-20 (PBST), then incubated with 50 μL of 1:5000 diluted goat anti-mouse HRP secondary antibody (Jackson ImmunoResearch) for 1 h. Plates were washed 3 times with PBST, and incubated with 50 μL TMB-Ultra (Thermo Scientific) for 15 min. The reaction was quenched with 50 μL 2 M H_2_SO_4_ and absorbance was read at 450 nm on a Tecan M200 plate reader.

### Tissue Collection, Cell Isolation, and Subtype Purification

After determination of significant titer for Ebolavirus VLPs (signal evident above background at dilution >1:10,000), mice were administered a final boost at day 77, and lymph nodes were collected 6 days later. For lymph nodes collection, mice were injected with 5–10 μL of 2% Evans Blue (Sigma) in PBS into the footpad. 30 min post-injection, mice were sacrificed by carbon dioxide asphyxiation followed by cervical dislocation. The skin and fur around the leg was removed to reveal the blue-stained popliteal lymph node (Fig. 1a), located just behind the knee. The lymph node was isolated and stored in PBS pH 7.4 supplemented with 0.1% (w/v) BSA, 2 mM EDTA in a 6-well plate (Corning). Lymph node was homogenized by mechanical disruption using two 18G needles. The cells were then passed through a 70 μm cell strainer (Corning), with additional disruption using the plunger from a 3 mL syringe to aid passage of single cells. Cells were then spun down at 500 g for 10 min in a swinging bucket rotor. The cell pellets were then resuspended in 2 mL red blood cell lysis buffer (155 mM NH_4_Cl, 12 mM NaHCO_3_, 0.1 mM EDTA) and incubated at room temperature for 3.5 min. The lysis reaction was quenched by adding 20 mL PBS buffer followed by centrifugation at 500 g for 10 min at RT. Cells were washed again with 5 mL PBS buffer and resuspended in a final volume of 1 mL buffer. Plasma cells were then isolated using the Miltenyi Plasma Cell Isolation kit (Miltenyi Biotec). Briefly, non-plasma cells were depleted by magnetic labeling of CD49b and CD45R followed by enrichment of magnetically-labeled CD138^+^ cells. CD45R is a pan-B cell marker expressed on naïve and activated B lymphocytes, but not on antibody-secreting cells. Conversely, CD138 is expressed on pre-B and immature B-lymphocytes in the bone marrow, lost upon emigration into secondary lymphoid tissues, and re-expressed upon differentiation into plasma cells.

### Single Cell V_H_:V_L_ Sequencing

Sorted cells were analyzed by single B cell V_H_:V_L_ sequencing as previously described^33^. Briefly, single cells were isolated into 125 pL wells printed in PDMS along with poly(dT) conjugated magnetic beads. Cell lysis and capture of mRNA was performed *in situ*, and beads were collected and emulsified to serve as template for emulsion overlap extension RT-PCR. A follow-up nested PCR resulted in 850 bp amplicons containing linked genetic information for V_H_ and V_L_ genes. 850 bp amplicons were analyzed using the Illumina MiSeq 2 × 250 platform. V_H_ and V_L_ genes were amplified separately for full-length V_H_ and V_L_ analysis using the Illumina MiSeq platform as previously described^33^.

### Sequence Analysis

Raw MiSeq data was analyzed as previously described^33^. Briefly, raw data were filtered for a minimum Phred quality score of 20 over 50% of nucleotides to ensure high read quality in the CDR3 regions of heavy and light genes. Sequence data were submitted to the IMGT information system for V-D-J germline gene mapping. Sequences were filtered for in-frame V-D-J junctions and V_H_:V_L_ pairs were compiled by exact CDRH3:CDRL3 nucleotide match. CDRH3 junction nucleotide sequences were clustered to 96% identity and resulting clusters with > = 2 V_H_:V_L_ reads were ranked by MiSeq read counts^33^. Due to read length limitations of current next-generation sequencing technology, the complete V_H_ and V_L_ genes were also sequenced and analyzed separately. Full-length V_H_ and V_L_ genes were filtered for a minimum Phred quality score of 20 over 50% of nucleotides and were compiled by CDRH3 and CDRL3 exact nucleotide match. Consensus sequences of V_H_ and V_L_ genes (i.e. from all reads passing quality filters and that contained exact matches to the CDRH3:CDRL3 pair of interest) were used for antibody gene synthesis, expression, and *in vitro* analysis^33^.

### IgG Synthesis, Expression, and Purification

Consensus V_H_ and V_L_ genes were designed and purchased as gBlocks (Integrated DNA Technologies) and cloned into the pcDNA3.4 vector (Invitrogen) containing *Oryctolagus cuniculus* IgG leader peptide as fusions to human IgG1 and kappa constant regions, respectively. Sequences of both the heavy and the light chain for each antibody variant were confirmed by Sanger sequencing. Plasmids for each antibody variant were transfected into Expi293 cells (Invitrogen) at a 1:3 heavy:light ratio. After incubating at 37 °C with 8% CO_2_ at 125 rpm for 6 days, the supernatant containing secreted antibodies was collected by centrifugation at 500 g for 15 min at 25°C. Supernatant was passed over a column of 0.5 mL Protein A agarose resin (Thermo Scientific) three times to ensure efficient binding. After washing with 20 column volumes of PBS, antibodies were eluted with 3 mL 100 mM citric acid pH 3.0 and immediately neutralized with 500 μL 1 M Tris pH 8.0. Antibodies were buffer exchanged into PBS, pH 7.4 utilizing Amicon Ultra-30 centrifugal spin columns (Millipore) for storage and subsequent use.

### ELISA

Costar 96-well ELISA plates (Corning) were coated with 50 μL of 4 μg/mL recombinant Ebola Glycoprotein (a gift from Dr. Erica O. Saphire, The Scripps Research Institute) or Ebola virus VLPs. The coated plates were incubated at 4 °C O/N, after which they were decanted and blocked with 2% milk in PBS for 2 h at RT. After blocking, 1:5 serially diluted antibodies were applied to the plates for 1 h, after which 1:5000 diluted donkey anti-human IgG HRP-conjuated secondary antibodies were applied (Jackson ImmunoResearch) for 1 h. For detection, 50 μL TMB-Ultra substrate was applied for 10 min before quenching with 50 μL 2 M H_2_SO_4_. Absorbance was measured at 450 nm using a Tecan M200 plate reader. Data were analyzed and fitted for EC_50_ using a 4-parameter logistic nonlinear regression model in the Prism software.

### Surface Plasmon Resonance

Antibody affinity to recombinant Ebola GP protein was measured by surface plasmon resonance using a BIAcore 3000 biosenor (Biacore). In order to fit the responses to 1:1 Langmuir binding model for more accurate affinity determination, antibodies were immobilized on the CM5 sensor chip (GE Healthcare) using the amine coupling chemistry. All binding experiments were done in HBS-EP buffer (10 mM HEPES pH 7.4, 150 mM NaCl, 3.4 mM EDTA, and 0.005% P20 surfactant) (GE Healthcare). GP was injected in triplicates at concentrations 80, 100, 200, 300, 400, 500, and 600 nM with a flow rate of 60 μL/min for 2 min and a dissociation time of 10 min. Regeneration of the antibody was performed by a single injection of 100 mM citric acid, pH 3.0. The response generated by flowing GP over a bovine serum albumin (BSA) coupled surface was used as control and was consequently subtracted. All kinetic parameters were determined in BIAevaluation 3.0 software and were reported as the average of three technical replicates.

### Wild type virus ELISA assays

All work with wild type virus was performed at BSL4 at Texas Biomedical Research Institute. All virus stocks were cultivated on Vero-E6 cells in DMEM with 2% FBS and antibiotics. When 80% of cells began showing a cytopathic effect, the culture supernatant containing virus was collected. Virus was purified as for VLPs by pelleting cell debris and then pelleting virus from the culture supernatants through 20% sucrose in 20 mM NaCl and 20 mM HEPES, pH 7.4. The virus pellets were resuspended in PBS and stored in aliquots at −80 °C until needed. Virus titers were determined by conventional plaque assay using Vero-E6 cells. For ELISA assays, an aliquot of virus was thawed and the equivalent of 10^6^ PFU of virus was diluted 1:3 into RIPA buffer. This was then diluted 1:100 into 10 mM sodium phosphate buffer, pH 7.4. After coating O/N, plates were washed with PBS containing 0.1% Tween-20 and incubated with each antibody starting at 1:100 of a 1 mg/mL stock and then over serial 4-fold dilutions on the plates. The secondary antibody was anti-human IgG HRP conjugate from Pierce. TMB Ultra substrate (Life Technologies) was used to detect antibody binding on plates. All assays were performed at least in duplicate and repeated 3 times. ELISAs were analyzed and fitted for EC_50_ using a 4-parameter logistic nonlinear regression model in the Prism software.

## Additional Information

**How to cite this article**: Wang, B. *et al.* Facile Discovery of a Diverse Panel of Anti-Ebola Virus Antibodies by Immune Repertoire Mining. *Sci. Rep.* **5**, 13926; doi: 10.1038/srep13926 (2015).

## Supplementary Material

Supplementary Information

## Figures and Tables

**Table 1:** List of characterized EBOV antibodies sequenced from PLN CD138^+^ cells.

**Mouse**	**Rank (Name)**	**CDRH3 Sequence**	**CDRL3 Sequence**	**Gene Usage**
1	1 (ZM1.1)	ARSFAY	QQSNEDPYTF	IGHV1-IGHJ3:IGKV3-IGKJ2
	2 (ZM1.2)	TGDGYYGFAY	FQGSHVPFT	IGHV6-IGHD2-IGHJ3:IGKV1-IGKJ4
	3 (ZM1.3)	ARGIGY	WQGTHFPFT	IGHV3-IGHJ3:IGKV1-IGKJ4
	4 (ZM1.4)	ARSTTATLDC	QQSDSWPTLT	IGHV14-IGHD1-IGHJ2:IGKV5-IGKJ5
	5 (ZM1.5)	ATISTATFPY	QQSDSWPTLT	IGHV1-IGHD1-IGHJ3:IGKV5-IGKJ5
	6 (ZM1.6)	ARRAMITTEGVDFDY	QQSRKVPWT	IGHV3-IGHD2-IGHJ2:IGKV3-IGKJ1
	7 (ZM1.7)	AREGYRYDWYFDV	QQRSSYPLT	IGHV1-IGHD2-IGHJ1:IGKV4-IGKJ5
2	1 (ZM2.1)	TRSVSDY	WQGTHFPHT	IGHV1-IGHD2-IGHJ2:IGKV1-IGKJ5
	2 (ZM2.2)	ARRTYRYDRFDY	QQWSSDPLT	IGHV1-IGHD2-IGHJ2:IGKV4-IGKJ5
	3 (ZM2.3)	TRRSNFPYYFDF	QQSIEDPFT	IGHV1-IGHD2-IGHJ2:IGKV3-IGKJ4
	4 (ZM2.4)	ARSELGATGFAY	QQGQSYPIFT	IGHV5-IGHD3-IGHJ3:IGKV15-IGKJ4
	5 (ZM2.5)	ARQKYGNYVLYWYFDV	QQWNSNPPT	IGHV5-IGHD2-IGHJ1:IGKV4-IGKJ4
	6 (ZM2.6)	TGMVTSY	LQHWNYPYT	IGHV6-IGHD2-IGHJ3:IGKV6-IGKJ2
	7 (ZM2.7)	VREGLGSYFDY	QQYYNYPRT	IGHV10-IGHD5-IGHJ2:IGKV8-IGKJ1

For each antibody, CDRH3:CDRL3 clonotypes and their V(D)J gene assignment are provided.

**Table 2:** SPR Binding kinetics and equilibrium dissociation constants (K_D_) towards uncleaved EBOV GP.

**Antibody ID**	**k**_**on**_ **(M**^**−1**^**s**^**−1**^)	**k**_**off**_ **(s**^**−1**^)	**K**_**D**_ **(nM)**
ZM1.1	(3.12 ± 0.55) × 10^4^	(1.16 ± 0.07) × 10^−3^	37.7 ± 0.55
ZM1.2	(1.17 ± 0.41) × 10^4^	(1.71 ± 0.12) × 10^−3^	156 ± 47.4
ZM1.3	(3.12 ± 0.53) × 10^4^	(2.39 ± 0.62) × 10^−4^	7.71 ± 1.77
ZM1.6	(3.95 ± 0.7) × 10^3^	(2.46 ± 0.12) × 10^−3^	635 ± 93.8
ZM2.1	(1.47 ± 0.23) × 10^4^	(2.97 ± 0.07) × 10^−4^	20.5 ± 3.44

Experiments were performed in three technical replicates and data were fit to a 1:1 Langmuir binding model.
