# Corrigendum: Facile Discovery of a Diverse Panel of Anti-Ebola Virus Antibodies by Immune Repertoire Mining

**DOI:** 10.1038/srep27229

**Published:** 2016-06-10

**Authors:** Bo Wang, Christien A. Kluwe, Oana I. Lungu, Brandon J. DeKosky, Scott A. Kerr, Erik L. Johnson, Hidetaka Tanno, Chang-Han Lee, Jiwon Jung, Alec B. Rezigh, Sean M. Carroll, Ann N. Reyes, Janelle R. Bentz, Itamar Villanueva, Amy L. Altman, Robert A. Davey, Andrew D. Ellington, George Georgiou

Scientific Reports 5: Article number: 13926; 10.1038/srep13926 published online: 09102015; updated: 06102016

Hidetaka Tanno and Chang-Han Lee were omitted from the author list in the original version of this Article. This has been corrected in the PDF and HTML versions of the Article.

The Author Contributions section now reads:

B.W., C.A.K., O.I.L., S.M.C., I.V., A.L.A., R.A.D. and G.G. designed the experiments. B.W., C.A.K., O.I.L., B.J.D., S.A.K., E.L.J., H.T., C.L., J.J., A.B.R., A.N.R., J.R.B. and R.A.D. performed the experiments. B.W., C.A.K., O.I.L., B.J.D., R.A.D., A.D.E. and G.G. analyzed the data. B.W., C.A.K., O.I.L., I.V., R.A.D., A.D.E. and G.G. wrote the manuscript.

