# Supplementary material for: Facile Discovery of a Diverse Panel of Anti-Ebola Virus Antibodies by Immune Repertoire Mining
Source: Sci Rep. 2015 Sep 10;5:13926. doi: 10.1038/srep13926 (PMC4564727; doi:10.1038/srep13926)
Supplement: Supplementary Information [file srep13926-s1.doc]

Bo Wang, Christien A. Kluwe, Oana I. Lungu, Brandon J. DeKosky, Scott A. Kerr, Erik L. Johnson, Jiwon Jung, Alec B. Rezigh, Ann N. Reyes, Sean M. Carroll, Janelle R. Bentz, Itamar Villanueva, Amy L. Altman, Robert A. Davey, Andrew D. Ellington, George Georgiou

**Facile Discovery of a Diverse Panel of Anti-Ebola Virus Antibodies by Immune Repertoire Mining**

**Supplementary Information**


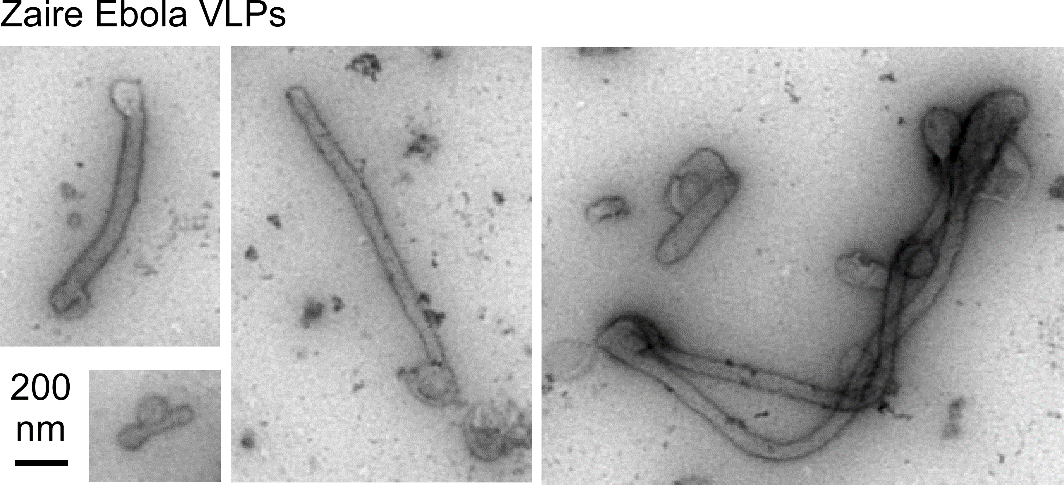

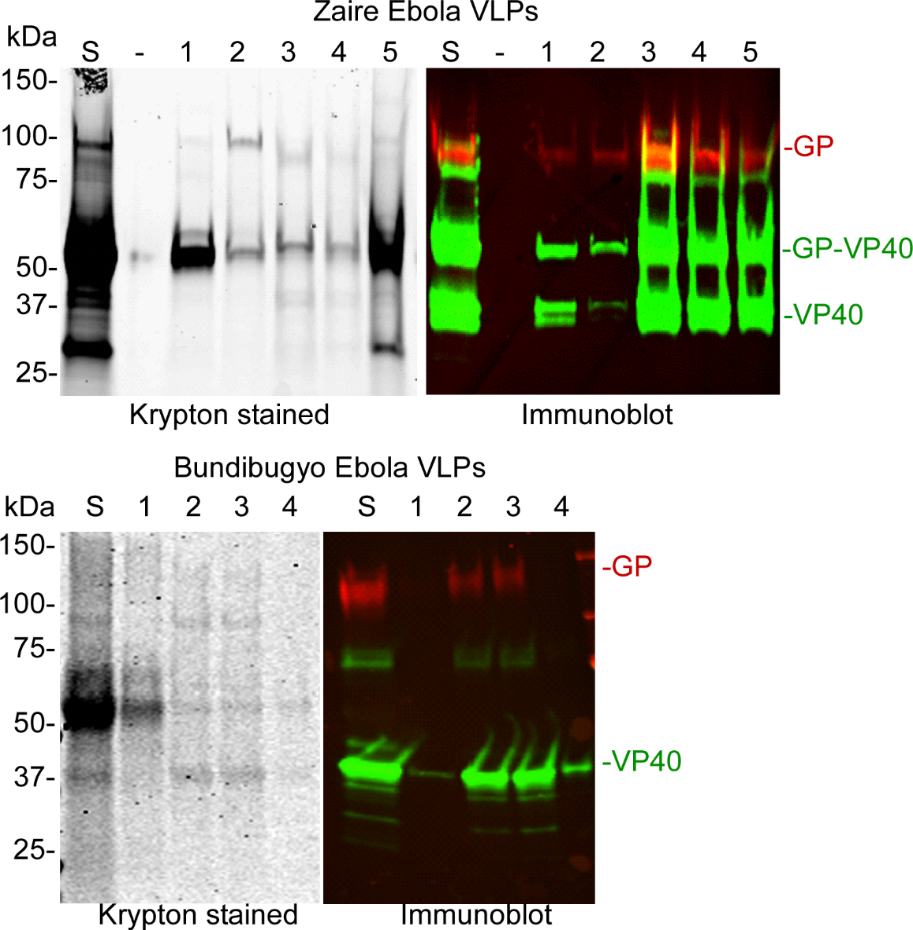


**Supplementary Figure 1: VLP production and characterization**. **(a)** Transmission electron microscopy images of EBOV VLPs. VLPs were purified by sucrose gradient and imaged using transmission electron microscopy. Particles of heterogeneous size were visible and examples are shown**. (b,c)** Total protein (left panel) and immunoblot (right panel) analysis of Ebola **(b)** and Bundibugyo **(c)** virus VLPs. VLPs were generated by transfecting plasmids encoding NP, VP40 and GP into 293FT cells. VLPs from culture supernatants were pelleted through sucrose and then applied to a sucrose gradient (S). Fractions were then collected from top (1-4) and material pelleting also collected (5). Gels were either stained with krypton total protein stain (left panels) or with VP40 (green) and GP (red) specific antibodies (right panels). Bundibugyo VLP pellet from the gradient was not analyzed.

**a**

**b**

**c**


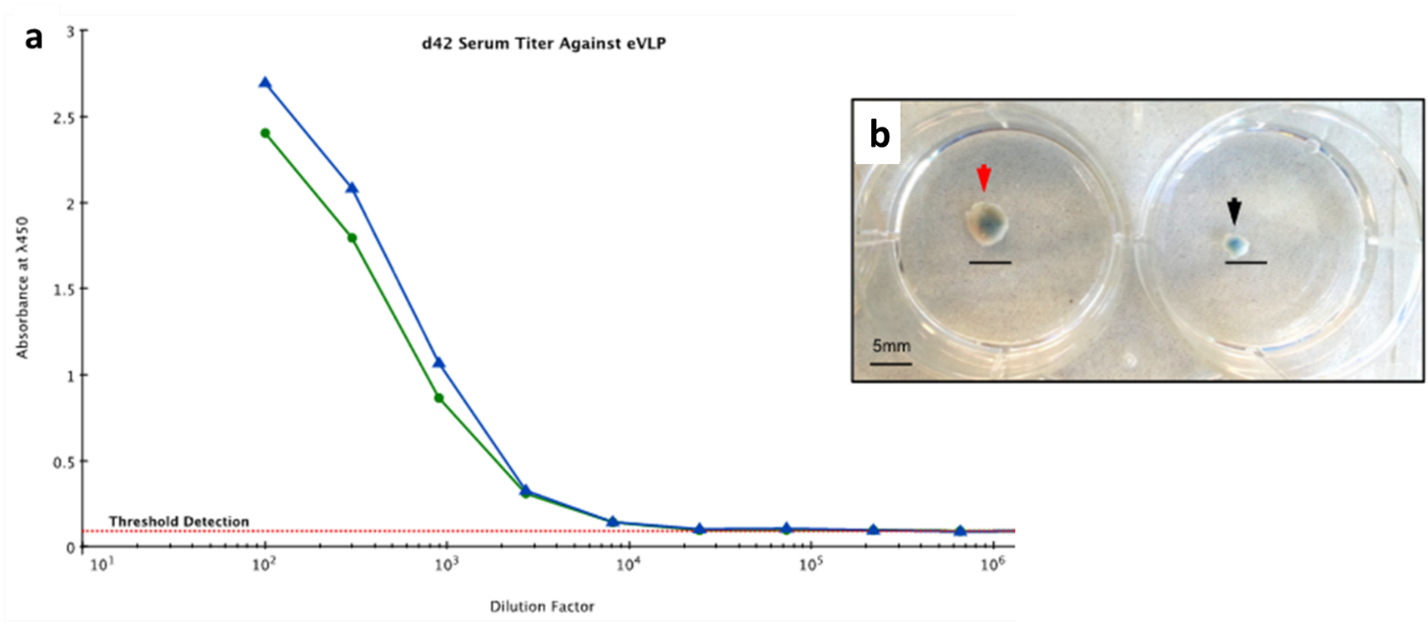


**Supplementary Figure 2: Footpad immunization with EBOV VLPs:** BALB/c mice were immunized with 5 µg Ebola VLPs in 10 µL PBS pH 7.4 emulsified in a 1:1 ratio with TiterMax Gold adjuvant with boosts on days 21 and 35. (**a**) Tail-vein bleeds were performed 7 days post day 35 boost, and antibody titers against VLPs measured in Mouse 1 (blue) and Mouse 2 (green). (**b**) Size comparison of the dissected ipsilateral (red arrow) and contralateral (black arrow) LNs. Ipsilateral LN: ~5mm in diameter; contralateral LN: ~2mm in diameter.


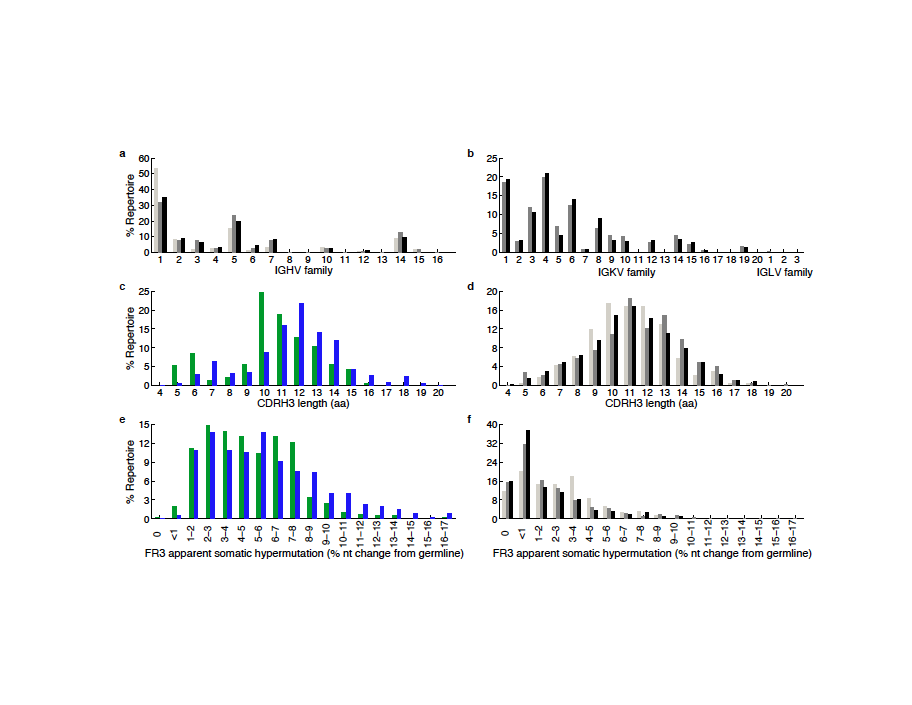


**Supplementary Figure 3: Characteristics of immune repertoires in CD138+ antibody secreting cells.** **(a,b)** V_H_ and V_L_ gene family usage of unique CDRH3 in previously published mice immunized with various antigens (Hen Egg Lysozyme: light gray; BRIGHT: dark gray; and C1s: black^1^). **(c)** CDRH3 length distribution of CD138^+^ PLN plasmablasts repertoire in EBOV VLPs immunized mice (mouse ZM1, green, mouse ZM2, blue) and; **(d)** CDRH3 distribution of the bone marrow plasma cell repertoire from mice immunized with Hen Egg Lysozyme (light gray); the transcription factor BRIGHT (dark gray); and the complement protein C1s (black) are shown for comparison^1^. **(e)** Apparent nucleotide mutation rate in the framework 3 (FR3) region of V_H_ of mouse ZM1 (green) and mouse ZM2 (Blue) PLN CD138^+^ plasmablasts repertoire; and **(f)** in mice immunized with various other antigens (Hen Egg Lysozyme: light gray; Bright: dark gray; and C1s: black^1^).

| **Antibody ID** | **Titer against VLP** | **Antibody ID** | **Titer against VLP** |
| --- | --- | --- | --- |
| **ZM1.1** | 312500 | **ZM2.1** | 312500 |
| **ZM1.2** | 2500 | **ZM2.2** | 100 |
| **ZM1.3** | 312500 | **ZM2.3** | 312500 |
| **ZM1.4** | 62500 | **ZM2.4** | 312500 |
| **ZM1.5** | 312500 | **ZM2.5** | 62500 |
| **ZM1.6** | 12500 | **ZM2.6** | 62500 |
| **ZM1.7** | 62500 | **ZM2.7** | 312500 |

**Supplementary Table 1: Antibody titers against EBOV VLPs.** Antibody titers were determined by calculating greatest dilution of 1 mg/mL antibody via ELISA that resulted in a signal of 3 standard deviations above background at 450 nm absorbance. Result is the mean of three technical replicates.


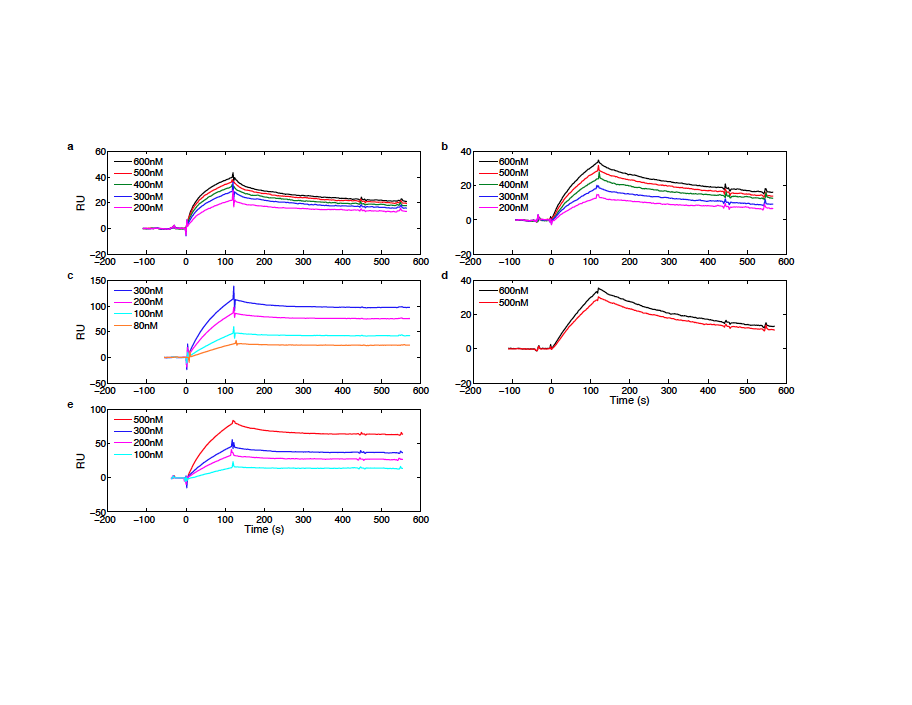


**Supplementary Figure 4:**  **BIACore sensorgrams for selected antibodies binding to recombinant EBOV GP:** (**a**) ZM1.1, (**b**) ZM1.2, (**c**) ZM1.3, (**d**) ZM1.6, (**e**) ZM2.1. Antibodies were immobilized on CM5 sensor chips and varying concentrations of GP were injected using a flow rate of 60 µL/min for 2 min. Experiments were performed in three technical replicates, and all curves were fit to a 1:1 Langmuir binding model using BIAevaluation software.

**Supplementary Figure 5: Phylogenetic relationships of Ebolavirus GP proteins.** Virus entries are listed as virus name, strain name, and year, followed by NCBI accession number. EBOV, Mayinga, 1976 as well as RESTV, Reston, 1996 VLPs were used as antigens for mouse immunizations. SUDV, Gulu, 2000; BDBV, Bundibugyo, 2007; EBOV, Kissidougou-C15, 2014; EBOV, 034-KS, 2008; and EBOV, Mayinga, N550K were selected for cross-reactivity studies. GP sequences were obtained from NCBI and aligned by neighbor joining using the Geneious software package.

##
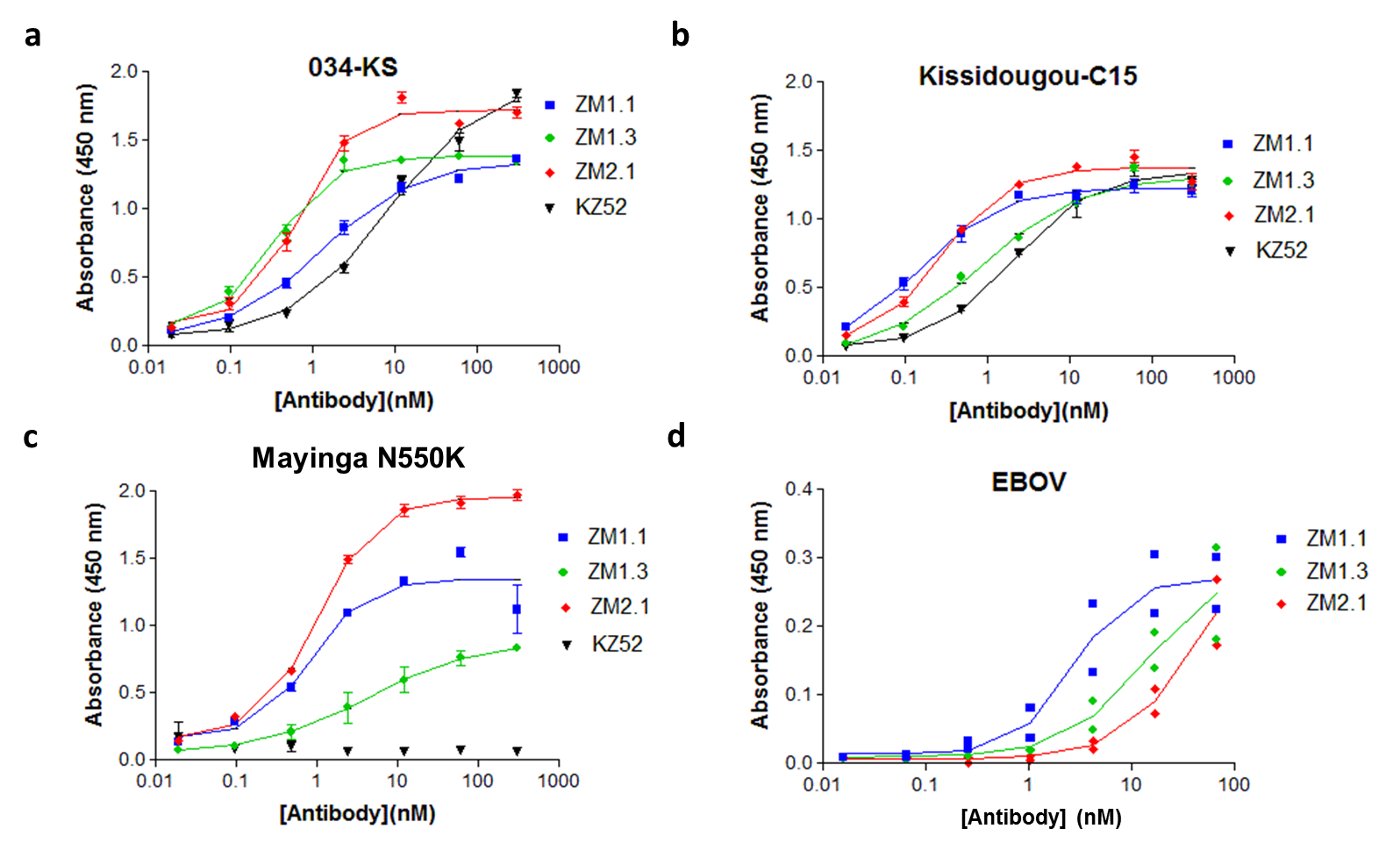


**Supplementary Figure 6**:  **Antibodies binding with varying specificity to selected Ebola VLP strains.** Antibodies were tested for binding to Ebola VLPs containing protein variants from two naturally occurring strains and one lab developed strain mutation of Ebola VLPs (**a-c**). Antibodies were tested for binding to 200 ng of (**a**) 034-KS strain VLPs (**b**) Kissidoughou-C15 strain VLPs and (**c**) Mayinga strain with GP mutation N550K VLPs. Antibody serial dilutions were performed in three technical replicates with error bars depicting standard error of the mean for replicates. Lines represent measurements fitted via 4-parameter logistic nonlinear regression for EC_50_. (**d**) Antibodies were tested for binding to live EBOV, Mayinga strain. Antibody serial dilutions were performed in two technical replicates. Lines represent measurements fitted via 4-parameter logistic nonlinear regression for EC_50_.

| Mouse | Rank (Name) | CDRH3  Sequence | CDRL3  Sequence | Gene Usage |
| --- | --- | --- | --- | --- |
| 2 | 4 (RM2.4) | ARWGWVGAMDY | QQSNSWPLT | IGHV5-IGHJ4:IGKV5-IGKJ5 |
| 3 | 2 (RM3.2) | ARSGGTN | WQGTHFPLT | IGHV1-IGHD4-IGHJ2:IGKV1-IGKJ5 |
|  | 3 (RM3.3) | ARHSPSYGNFAWFAY | QQWSNPPWT | IGHV5-IGHJ3:IGKV4-IGKJ1 |

**Supplementary Table 2. List of characterized RESTV antibodies sequenced from PLN CD138^+^ cells binding to RESTV GP.** For each antibody, CDRH3:CDRL3 clonotypes and their V(D)J gene assignment are provided.

|  | EBOV binding | BDBV binding | SUDV binding |
| --- | --- | --- | --- |
| ZM1.1 | + | - | - |
| ZM1.2 | - | - | - |
| ZM1.3 | + | - | - |
| ZM1.4 | + | + | - |
| ZM1.5 | + | - | - |
| ZM1.6 | + | - | - |
| ZM1.7 | + | + | + |
| ZM2.1 | + | - | - |
| ZM2.2 | + | + | + |
| ZM2.3 | + | + | - |
| ZM2.4 | NA | NA | NA |
| ZM2.5 | + | + | + |
| ZM2.6 | + | + | + |
| ZM2.7 | + | - | - |
| KZ52 | + | - | - |

**Supplementary Table 3:**  **Binding of antibodies isolated via mining of the PLN repertoire to live EBOV, BDBV, and SUDV.** ELISA assays were conducted by binding serial dilutions of antibodies to viruses resuspended and diluted in RIPA denaturing buffer. Antibody serial dilutions were performed in two technical replicates.

**Supplementary References**

1. Reddy, S. T*. et a*l. Monoclonal antibodies isolated without screening by analyzing the variable-gene repertoire of plasma cells*. Nat. Biotechno*l**. 2**8, 965–969 (2010).
